# Playground lead levels in rubber, soil, sand, and mulch surfaces in Boston

**DOI:** 10.1371/journal.pone.0216156

**Published:** 2019-04-25

**Authors:** Khaled S. Almansour, Nicholas J. Arisco, May K. Woo, Anna S. Young, Gary Adamkiewicz, Jaime E. Hart

**Affiliations:** 1 Department of Environmental Health, Harvard T.H. Chan School of Public Health, Boston, Massachusetts, United States of America; 2 Channing Division of Network Medicine, Department of Medicine, Brigham and Women’s Hospital and Harvard Medical School, Boston, Massachusetts, United States of America; East Carolina University, UNITED STATES

## Abstract

Rubber surfacing is often used in playgrounds due to its potential injury prevention benefits and as a way to recycle waste tires. Available research on chemicals in recycled rubber has focused on synthetic turf applications, but is limited for playground rubber surfacing. Potential lead contamination from vulcanizing agents used in rubber surfacing are a possible concern; however this has not been researched. We examined levels of lead in poured-in-place rubber and compared them to levels in soil, sand, and wood mulch materials from 28 randomly selected playgrounds in Boston, MA, USA using X-ray fluorescence. To evaluate the association between material type and lead concentrations, we conducted a two-way ANOVA with repeated measures and built a linear regression model controlling for distance to major roadway, neighborhood-level status as an environmental justice area, peeling paint on the playground, and rubber condition. Average lead levels were 65.7 μg/g for soil, 22.0 μg/g for rubber, 8.5 μg/g for sand, and 9.0 μg/g for mulch. Our finding of lower concentrations of lead in sand and mulch compared to rubber and soil should be used to inform playground design to optimize children’s health, alongside other chemical and safety considerations.

## Introduction

Rubber surfacing has become more widespread in playground design due to its potential injury prevention benefits [1–3]. From 2005 to 2015, the amount of ground tire manufactured for playground use rose from 19,000 to approximately 225,000 tons [4]. Several types of playground surfaces are constructed from tire rubber: rubber tiles, poured-in-place rubber, bonded rubber, loose-fill rubber mulch, and synthetic turf [5]. In addition to its shock-absorbent qualities, rubber surfacing is also a useful and environmentally sustainable application for recycled waste tires. Roughly 300 million auto tires are disposed of in the United States every year, and are often restricted from landfills to reduce the potential for tire fires and mosquito breeding habitats [3, 6–8].

There is growing public concern about the toxicity of recycled tire rubber materials used as infill in synthetic turf athletic fields. Limited research thus far has found that crumb rubber sometimes contains harmful polyaromatic hydrocarbons, phthalates, and benzothiazole [5, 9–11]. Some investigations also found harmful levels of lead in crumb rubber and turf fiber samples from synthetic turf fields, sometimes exceeding the 400 μg/g federal limit for lead in soil in play areas [12–17]. Historically, lead oxide was used as a vulcanizing agent in the manufacturing process to make tire rubber more elastic. Lead oxide has since been replaced by zinc oxide as the primary vulcanizing agent, but studies demonstrate that this alternative still contains lead contamination [5,18]. However, no known published studies have investigated lead levels in the other types of rubber surfacing used in playgrounds in the United States, and playground lead exposures remain in question [5,19].

Children at playgrounds are especially susceptible to lead exposure and the neurotoxic effects of lead due to their early stage of development, higher absorption rates, and high exposure risk behaviors such as frequent ground contact and hand-to-mouth behavior [20]. Children can be exposed to lead from rubber material through dermal contact, ingestion, or inhalation [5], and risk of exposures should be minimized [21].

The EPA has suggested sand and wood mulch as alternatives to rubber materials in athletic fields and playgrounds that potentially have lower chemical exposures but have the necessary injury prevention properties such as shock absorbance for falls [22,23]. However, no known studies have specifically compared lead or other heavy metals concentrations between different surfacing materials in playgrounds. Studies have indicated that lead has demonstrated to adsorb to the smaller soil fractions (e.g., silt, clay) [24, 25]; therefore, heavy metals may adsorb less readily to surfacing materials with larger size fraction particulates such as sand or wood mulch. This study aims to evaluate levels of lead in rubber surfacing compared to sand, wood mulch, and soil within playgrounds in Boston, Massachusetts.

Boston, Massachusetts was chosen as the study area for accessibility reasons and previous indication of high-risk communities within Boston for elevated blood lead levels. From 2009 to 2013, the neighborhoods of North Dorchester, Roxbury and Mission Hill, and East Boston contributed over 50% of incident cases of blood lead levels exceeding 5 μg/L, indicating that these neighborhoods with higher rates of poverty and minority populations make up a disproportionate number of children with elevated blood lead levels [26]. In our analyses, we will also test the hypothesis that distance from playground to major roadway could increase lead loading in ground materials due to historic leaded gasoline emissions [27, 28]. While the phase-out of leaded gasoline in the 1980s led to a 99% decrease in lead concentrations in the air, the environment still may contain a reservoir of lead retained soil and dust [29–31].

## Materials and methods

### Site selection

We selected a total of 28 playgrounds from the City of Boston’s list of public parks and playgrounds based on equal distribution of median income as a socioeconomic status (SES) indicator as reported by the Boston Public Health Commission [32, 33]. To obtain equal distribution of playgrounds among SES levels, we randomly selected 9 playgrounds from each of three neighborhood-level SES categories. To do so, we classified neighborhoods as “low SES” if they had an average median household income below the Boston 2006–2010 average median household income, “medium SES” if they had an average median household income within the Boston 2006–2010 average median household income, and “high SES” if they had an average median household income above the Boston 2006–2010 average median household income [34]. Only playgrounds with at least two material types were included in the analysis. Although all had soil, one of these 27 playgrounds did not have rubber, so we performed random selection of one additional site within the same SES classification to achieve at least nine samples of both rubber and soil within each SES level.

### Data collection

We collected rubber, soil, sand, and mulch materials from each playground using plastic spoons and bags. At each site, we collected two samples each for mulch, soil, and sand, at opposite ends of the playground when possible. For rubber samples, we collected one to three samples when easily extractable. When unable to collect rubber samples for off-site testing (four playgrounds), we performed X-ray fluorescence (XRF) measurements on-site. When sand was not within the confines of the playground (six playgrounds), we collected sand samples from immediately adjacent baseball fields as proxies. We also recorded geographic coordinates of the sampled sites, the type and condition of the rubber surfacing, the presence/absence of peeling paint on playground structures, the number of painted buildings immediately surrounding the playground, and the number of those buildings with visibly peeling exterior paint. While age or year of last renovation of the playgrounds and surrounding homes were not available, observations of peeling paint on playground structure was used as a proxy for playground age which may contribute to lead concentrations in the surface materials. Observations of surrounding building paint conditions were taken as potential lead dust contribution, but the distribution of observations was not adequate for assessing it as a contributor. Playgrounds were sampled over four days between October and November 2017.

We measured samples for lead with a handheld Niton^TM^ XL3t XRF Analyzer (ThermoFisher Scientific). Measurements were taken for 90 seconds in soil mode. Sand and soil samples were measured once, and mulch and rubber measurements were repeated once or twice due to the material’s heterogeneous nature. For the rubber samples, both the top (surface) and underside were measured.

We calibrated the XRF regularly during sampling against three soil standard references ranging in lead concentrations (NCS DC 73308 (27±2 μg/g), GBW 07411 (2700±100 μg/g Pb), and RCRA (500±100 μg/g Pb) and two blanks (SiO_2_ blank and a clean, empty plastic bag). The lead levels fell within the 95% confidence intervals for lead for 100% of SiO2 (n = 25), plastic bag (n = 29), RCRA (n = 26), and GBW (n = 25) standard measurements. For 76% of NCS measurements (n = 25), the lead levels fell within the 95% confidence intervals, while the upper bounds of the remaining measurements were, on average, 2.7 μg/g lower than the standard’s given range. Based on these readings, the XRF’s lead measurements can be considered conservative.

To ensure that the measurements were not affected by slight variations in moisture content, we randomly selected one sand, one mulch, and two soil samples from each sampling day to undergo complete dehydration. We found no statistically significant differences between the lead measurements of the original samples and the samples post-drying, and therefore continued our measurements on undried samples.

To obtain distance-to-roadway data, we collected roadway information from 2013 Massachusetts Department of Transportation Inventory Data [35]. We defined major roadways to include interstate highways, U.S. highways, state routes, and major roads (arterial and connectors) as available. The distance from the GPS coordinates of our samples to the nearest major roadway was calculated for each playground and sample in ArcGIS 10.3 (ESRI, Redlands, CA). To collect information on neighborhood-level SES factors that might be associated with nearby lead sources (such as industry or leaded paint), we accessed environmental justice (EJ) index data available from the United States 2010 Census data at the block group level and identified the EJ index of the block group that the playground resides in using ArcGIS. The EJ index as established by the Massachusetts Executive Office of Energy and Environmental Affairs (EOEA) was based on the number of the following criteria met by that block group’s population: low median income (median income less than or equal $40,673), high percent minority (greater than or equal to 25% minority), and high non-English speaking population (greater than or equal to 25% of households identifying as English-isolated), and ranged from 0–3 [36]. As the chosen sites were all public playgrounds in Boston, specific permission to access the locations for the field studies was not required. The sampling of materials at the sites did not involve endangering or otherwise disturbing protected species or park conditions.

### Data analysis

From 28 playgrounds, we collected 85 unique site and sample type combinations. For our analysis, we used average lead levels for each material within a playground as our outcome of interest. There was no missing data on our outcome of interest nor our predictors: playground material type, distance to major roadway, and environmental justice criteria. We coded measurements below the limit of detection (LOD) as half of this value (6.5 μg/g Pb). The number of observations for each material type varied due to different surfaces present at the selected sites. We tested all variables to be included in the model for normality using the Shapiro-Wilk test and visualization of histograms. Average lead levels and distance to roadway were both non-normally distributed with p<0.001. To achieve normality, we performed a square root transformation for distance to roadway.

To test for the statistical differences in average lead levels across playground material types, we employed two-way analysis of variance (ANOVA) with repeated measures to account for dependence of samples within the same site. We used average lead levels as our outcome and material type categorized as mulch, soil, rubber, and sand as our predictor. To correct for violation of the sphericity assumption, we used a Greenhouse-Geisser p-value correction. In order to reduce risk of multiple testing and false positives, we first examined the p-value for this global test of significance and only moved forward with pairwise comparisons of material types when the global p-value was less than α = 0.05. We then employed a post-hoc Games-Howell Test which allows for dependence of samples and unequal sample sizes between categories.

To evaluate specific differences in lead concentrations between material types while adjusting for possible confounding variables, we built a linear regression model with lead concentrations as our outcome. Soil was chosen as the reference group for our categorical variable, material type, because it had the highest number of samples and least number of measurements below LOD. Variables included in the multiple regression model were chosen *a priori* based on evidence in the scientific literature indicating potential contribution to lead concentrations in the environment. We included proximity to major roadway from the playground area as a potential confounder to this association. We collapsed environmental justice criteria to a binary variable, with meeting no EJ criteria as the reference value and meeting at least one EJ criteria as the comparison value. Other predictors in the model included observed presence of peeling paint on playground structures and condition of the rubber. Observed peeling paint on playground structures was used as a binary variable for presence versus absence, and rubber condition was used as a categorical variable for high deterioration and some deterioration, with no deterioration as the reference group. We did not include presence of nearby painted buildings nor peeling paint on these buildings as covariates because of limited occurrence (n = 2). Our final model was:

$$Average\;Pb=\;\beta_{1}+\beta_{2}*Mulch+\;\beta_{3}*Rubber+\;\beta_{4}*Sand+\;\beta_{5}*\sqrt{Distance\;to\;roadway}+\;\beta_{6}*EJArea+\;\beta_{7}*PeelingPaintPresent+\;\beta_{8}*RubberHighDeterioration+\;\beta_{9}RubberSomeDeterioration+\;\varepsilon$$

Where *EJArea* denotes if the playground location meets any of the EJ criteria, *PeelingPaintPresent* denotes if there is peeling paint in the playground, *RubberHighDeterioration* denotes if there is highly damaged rubber in the playground, and *RubberSomeDeterioration* denotes if there is slightly damaged rubber in the playground. We were interested in assessing p-values for all three material types compared to soil, so we incorporated a Bonferroni adjustment of 3 to reduce the chance of false positives. Thus, significance was assessed at the α = 0.05/3 = 0.017 level. We used conservative, robust standard errors for our model to account for heteroskedasticity in the geometric mean lead levels.

We tested the importance of cross-contamination of materials at playgrounds by conducting a two-sample t-test assuming unequal variances for the difference in lead levels between underside measurements of rubber samples and topside measurements of rubber samples. Naturally, we would expect the underside measurements not to have cross-contamination from soil or other materials, whereas the topside measurements could have had cross-contamination. Only two rubber samples had missing data on sides measured, but both measurements for each of these samples were below the LOD.

To test for cross-contamination of soil in rubber, sand, and mulch samples, we ran Spearman tests for the correlation between the average lead levels in soil versus rubber, sand, and mulch within playgrounds. We conducted all statistical analyses in R (version 3.3.3).

## Results

Baseline prevalence of the covariates of interest are summarized in Table 1 and Table 2 shows summary statistics for average lead levels measured in each material type. Sand had, on average, the lowest lead levels while soil had, on average, the highest lead levels. Rubber had the second highest average lead level. The highest average lead level in any playground was 336.6 μg/g in soil. Among all the non-averaged sample measurements, one soil sample reached 613 μg/g lead. All other measurements ranged from below LOD to 217.1 μg/g. Nine playgrounds had a soil sample greater than 80 μg/g and two playgrounds had a rubber sample greater than 80 μg/g.

**Table 1:** Characteristics of sampled playgrounds, Boston, 2017 (n = 28).

Characteristic		No. (%)
**Sample type present**	**Rubber**	**27 (96.4)**
	**Soil**	**28 (100)**
	**Sand**	**15 (53.6)**
	**Mulch**	**15 (53.6)**
**Neighborhood-level SES**	**Low**	**9 (32.1)**
	**Medium**	**9 (32.1)**
	**High**	**10 (35.7)**
**Observed rubber deterioration**	**None**	**11 (39.3)**
	**Low**	**5 (17.9)**
	**High**	**11 (39.3)**
**Observed peeling paint on play structure**	**No**	**17 (60.7)**
	**Yes**	**11 (39.3)**
**Number of homes with peeling paint directly surrounding playground**	**0**	**26 (92.9)**
	**1–2**	**2 (7.1)**
**Environmental justice criteria met**	**0**	**12 (42.9)**
	**1**	**12 (42.9)**
	**2**	**2 (7.1)**
	**3**	**2 (7.1)**
**Distance to nearest major roadway**	**≤ 100m**	**17 (60.7)**
	**101-200m**	**8 (28.6)**
	**201-300m**	**3 (10.7)**

Summary of observed characteristics of playgrounds sampled in 2017 in Boston, MA.

**Table 2:** Lead concentrations by material type (μg/g).

	Soil	Rubber	Sand	Mulch
Min	12.66	5.96	5.85	4.60
Max	336.60	73.19	22.98	26.15
Median	41.85	11.68	7.03	6.70
Mean	65.66	21.97	8.53	9.05
SD	68.37	20.6	4.45	5.99

Summary statistics for lead levels (μg/g) by material type from 28 playgrounds sampled in Boston, 2017.

**Fig 1:**
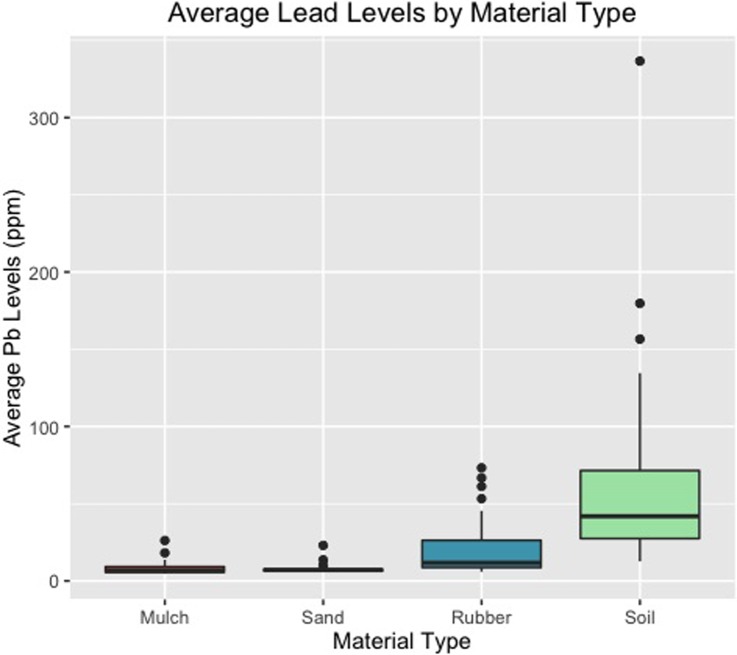
Average lead levels by material type. Average lead levels (ppm or μg/g) by material type (mulch, sand, rubber, and soil).

The average lead levels by material type are displayed with boxplots (Fig 1). Results from the two-way ANOVA with repeated measures indicated statistically significant differences between the average lead levels in the four playground material types (p = 0.026). Because this test showed significance, we proceeded with the post-hoc Games-Howell Tests for pairwise comparisons with unequal sample sizes and dependence. For lead values, all material type pairs were statistically significant at the 0.05 level, except for sand-mulch (Table 3). Soil average lead levels were significantly higher than rubber, sand, and mulch, while rubber average lead levels were significantly higher than sand and mulch.

**Table 3:** Differences in average lead concentrations for each material pair.

Pair	Difference in Pb, μg/g (95%CI)	P-value
**Rubber—Mulch**	12.92 (1.41, 24.43)	0.023*
**Sand—Mulch**	-0.53 (-5.81, 4.76)	0.993
**Soil—Mulch**	56.61 (21.06, 92.16)	0.001*
**Sand—Rubber**	-13.45 (-24.67, -2.23)	0.014*
**Soil—Rubber**	43.69 (7.07, 80.30)	0.014*
**Soil—Sand**	57.13 (21.67, 92.60)	0.001*

Difference in average lead concentrations (μg/g), with 95% confidence intervals, for each material pair.

* The two materials are statistically significantly different (p<0.05) based on the Games-Howell post-hoc pairwise test.

The results of the linear regression model for the association between material type and lead concentration, adjusted for the playground neighborhood’s identification as an environmental justice neighborhood, the distance to nearest major roadway, the presence of peeling paint on the playground, and the rubber condition, are summarized in Table 4.

**Table 4:** Results for the multivariable regression model.

Material Type	β	95% CI for β	Δ Average Pb	95% CI for Δ Average Pb
**Soil (Reference)**	55.00	(25.25, 84.76)	N/A	N/A
**Rubber**	-55.97*	(—83.17, -30.77)	-49%	(-73%, -23%)
**Sand**	-42.56*	(-64.56, -50.57)	-56%	(-80%, -28%)
**Mulch**	-58.23*	(-84.57, -31.38)	-49%	(-73%, -23%)

Results for the multivariable regression model for the association between playground material type (soil, rubber, sand, and mulch) and lead concentrations, adjusting for neighborhood-level SES as indicated by identification as an environmental justice area, distance to major roadway, presence of peeling paint in the playground, and rubber condition.

* Average lead levels for this material significantly different than soil levels (p<0.001).

After controlling for other covariates, rubber had 49% (95%CI: {-73%,-23%}) less lead on average compared to soil (p<0.001) while sand and mulch had 56% (95%CI: {-80%,-28%}) and 49% (95%CI: {-73%, -23%}) less lead than soil on average (sand p<0.001; mulch p<0.001). None of the covariates in our model were significant at the α = 0.05 level. The model R^2^ was 0.3266.

In evaluating the contribution of cross-contamination of materials affecting rubber lead levels, the two-sample t-test suggested that there is no difference in the mean lead levels between measurements of rubber samples taken from the underside and the top (p = 0.26). In addition, Spearman correlation tests between lead levels in soil and lead levels in rubber, sand, or mulch from the same site were not statistically significant (p = 0.40, p = 0.79, and p = 0.46, respectively). Thus, we are confident that soil lead levels were not correlated to the rubber, sand, and mulch lead levels measured in the same playground.

## Discussion

Our sample of 28 playgrounds in Boston demonstrated that sand and mulch had lower lead concentrations compared to poured-in-place rubber and soil. Soil lead concentrations (mean 65.66 μg/g) were typical for Massachusetts [37]. At our sample sites, soil was typically found on the edges of playgrounds, outside main play areas. As a result, children may not be exposed to soil lead with as much frequency as the other materials assessed. Rubber surfaces were commonly found directly underneath and around play structures. Rubber lead concentrations were similar in range compared to those found in recent crumb rubber infill samples from synthetic turf surfaces in New York City (<LOD to 100μg/g) [14,38–39]. One soil sample did exceed the EPA soil limit of 400 μg/g and several other soil and rubber samples exceeded the California residential soil guideline of 80 μg/g [19,40]. Considering that our samples were taken from playgrounds where children as young as six months old play, however, lead concentrations should be minimized in all playground materials. The high variability in the lead concentrations in soil and rubber samples relative to the sand and mulch may indicate local contamination at certain sites but may also be an artifact of the higher number of samples collected for these materials (n = 27 for rubber and n = 28 for soil, while sand and much had n = 15 each). Further sampling would be necessary to determine the nature of the variability within material type in further detail.

One study found that lead in two samples of synthetic turf rubber infill was bioaccessible in synthetic gastric fluid, but more research is needed to evaluate the bioaccessibility of rubber material from inhalation and dermal exposure routes [38]. In addition, studies have found a relationship between lead concentrations in soil and dust at playgrounds and on the hands of children playing, but further research is needed to investigate the quantity of lead from both deteriorated and non-deteriorated rubber surfacing on playgrounds that is taken up by children [41–42]. Various physical and chemical substrate properties such as pH, organic matter content, texture, and porosity may influence the bioavailability and uptake of lead from the environment and would be essential to consider when assessing and comparing exposures from the different materials [43]. Furthermore, the detected concentrations in the current study does not necessarily reflect the concentrations in the material’s topmost surface which would be of relevance for refining an exposure estimate.

Our results should be interpreted as a pilot investigation of playground materials as there were limitations in our study design. A larger sample size of playgrounds spanning a larger geographic domain would add more power and increase generalizability. However, our approach to sample from an equal number of playgrounds in low, medium, and high SES provides a level of generalizability to our results. Second, sample collection of playgrounds where many different materials are present in a small area introduces the issue of cross-contamination, particularly soil in mulch samples. However, careful sampling was performed to collect isolated samples, and repeated XRF measurements for each material were taken to account for potential heterogeneity and contamination. We found no significant difference in lead concentrations between the underside and top surface of the crumb rubber samples, indicating no contamination of rubber samples by soil or dust. Together, these precautions helped minimize measurement error due to contamination. Third, the indicators for SES (income and EJ criteria) were limited in their geographic resolution and did not necessarily represent the population of which the playground serves.

We also faced precision and detection limit limitations with the XRF analyzer. There were 5 playgrounds with a soil sample below the LOD for lead, 18 with a rubber sample below the LOD, 14 with a sand sample below the LOD, and 15 with a mulch sample below the LOD. However, only 5 playgrounds had all rubber measurements below the LOD. For these measurements, the median detection limits were 14.2 μg/g for soil, 13.4 μg/g for rubber, 13.4 μg/g for sand, and 11.8 μg/g for mulch. The recoding of data points below LOD as half the limit of detection could have introduced bias into our results. In further studies, sampling equipment with a lower limit of detection would be preferable. In addition, the XRF analyzer used in the present analysis may be optimized to measure metal levels in soil and sand but not materials with different physical characteristics such as rubber and mulch. However, replicate measurements for these samples were taken to help account for this limitation.

Our study also had many strengths. This was the first study that we know of to compare lead levels across material types in playgrounds. Our study captured a relatively large percent (22%) of listed Boston public playgrounds, and nearly all playgrounds sampled in the present study (96%) had both soil and rubber surfacing. All of the rubber materials we sampled appeared to be of the same poured-in-place type. While our study design incorporated a range of playground conditions in neighborhoods of varying levels of SES representative of Boston’s diverse population distribution, further studies would be necessary to produce robust results that could be generalizable to other playgrounds in Boston and beyond.

Our results and study limitations suggest a need for further research to compare metal and other chemical exposure levels across playground materials using larger sample sizes, a wider geographic domain, and with more precise analysis methods for specific material types. While these results present environmental levels of lead, extending these results to an evaluation of dose and risk to children would be important, as the exposure pathways and bioavailability of lead within a rubber matrix would be expected to differ from lead in soil, for example.

Use of wood mulch and sand as primary playground materials or as ground covers on top of lead-contaminated soil could help prevent soil lead exposures [41,44]. In addition, wood mulch may be more effective at preventing injuries than some rubber surfaces, but more research is needed. One recent study of California playgrounds found that only a third of 32 rubber surfaces passed impact attenuation tests for state-mandated head impact criteria, whereas all five wood chip surfaces passed [45].

In conclusion, the relative safety of sand and mulch compared to soil and rubber in regards to lead exposure should be considered in playground design alongside other safety factors such as other potential chemical exposures and performance in injury prevention.

## Supporting information

S1 File Results of sampled playgrounds. Demographic data, sampled concentrations, and observations for the 28 playgrounds sampled in Boston, MA, USA.
(CSV)

S2 File Results of playground rubber samples. Detailed data on XRF sampled concentrations for the top versus underside of playground surface rubber samples.
(CSV)

## References

[1] Mott A, Rolfe K, James R, Evans R, Kemp A, Dunstan F, et al Safety of surfaces and equipment for children in playgrounds. The Lancet 1997 28 6 1997;349(9069):1874–1876. 10.1016/S0140-6736(96)10343-3 9217759

[2] Norton C, Nixon J, Sibert JR. Playground injuries to children. Archives of disease in childhood. 2004 2 1;89(2):103–8. 10.1136/adc.2002.013045 14736615 PMC1719797

[3] Caldwell J. Tire-Derived Product (TDP) Descriptions and Case Studies Playground Surfaces. 2016; Available at: www.calrecycle.ca.gov/tires/products/Types/Playground.htm. Accessed December 10, 2017.

[4] Rubber Manufacturers Association. 2015 U.S. Scrap Tire Management Summary. 2016 Abelsohn AR, Sanborn M. Lead and children: Clinical management for family physicians. Can Fam Physician 2010 06;56(6):531–535. PMC2902938 20547517

[5] U.S. Environmental Protection Agency, Centers for Disease Control and Prevention / Agency for Toxic Substances and Disease Registry, U.S. Consumer Product Safety Commission / Directorate for Health Sciences. Federal Research Action Plan on Recycled Tire Crumb Used on Playing Fields and Playgrounds. 2016;16(364).

[6] U.S. Environmental Protection Agency Office of Solid Waste. Markets for Scrap Tires. 1991;EPA/530-SW-90-074A.

[7] Massachusetts Department of Environmental Protection Bureau of Waste Prevention. Best Management Practices for Automotive Recyclers. 2006(2) Bleyer A. Synthetic Turf Fields, Crumb Rubber, and Alleged Cancer Risk. Sports Medicine 2017:1–5. 10.1007/s40279-017-0735-x 28493060

[8] Claudio L. Synthetic turf: health debate takes root. Environ Health Perspect 2008 3;116(3):A116–22. 10.1289/ehp.116-a116 18335084 PMC2265067

[9] Llompart M, Sanchez-Prado L, Lamas JP, Garcia-Jares C, Roca E, Dagnac T. Hazardous organic chemicals in rubber recycled tire playgrounds and pavers. Chemosphere. 2013 1 31;90(2):423–31. 10.1016/j.chemosphere.2012.07.053 22921644

[10] Celeiro M, Lamas JP, Garcia-Jares C, Dagnac T, Ramos L, Llompart M. Investigation of PAH and other hazardous contaminant occurrence in recycled tyre rubber surfaces. Case-study: restaurant playground in an indoor shopping centre. International Journal of Environmental Analytical Chemistry. 2014 9 26;94(12):1264–71.

[11] Highsmith R, Thomas K, Williams R. A Scoping-Level Field Monitoring Study of Synthetic Turf Fields and Playgrounds 2009 National Exposure Research Laboratory, U.S. Environmental Protection Agency.

[12] Van Ulirsch G, Gleason K, Gerstenberger S, Moffett DB, Pulliam G, Ahmed T, et al Evaluating and Regulating Lead in Synthetic Turf. Environ Health Perspect 2010 8/27;118(10):1345–1349. 10.1289/ehp.1002239 20884393 PMC2957910

[13] Pavilonis BT, Weisel CP, Buckley B, Lioy PJ. Bioaccessibility and risk of exposure to metals and SVOCs in artificial turf field fill materials and fibers. Risk Analysis 2014;34(1):44–55. 10.1111/risa.12081 23758133 PMC4038666

[14] NYC Department of Parks and Recreation. Synthetic Turf Lead Results. 2008; Available at: https://www.nycgovparks.org/news/reports/synthetic-turf-tests. Accessed December 12, 2017.

[15] Kim S, Yang JY, Kim HH, Yeo IY, Shin DC, Lim YW. Health risk assessment of lead ingestion exposure by particle sizes in crumb rubber on artificial turf considering bioavailability. Environ Health Toxicol 2012;27:e2012005 10.5620/eht.2012.27.e2012005 22355803 PMC3278598

[16] Simcox N, Bracker A, Meyer CJ. Artificial Turf Field Investigation in Connecticut Final Report. 2010.

[17] U.S. Environmental Protection Agency. Federal Register Part III Lead; Identification of Dangerous Levels of Lead; Final Rule. 2001;40 CFR(Part 745):1206.

[18] California OEHHA. Final Report: Revised California Human Health Screening Levels for Lead and Beryllium 2009.

[19] Reichelderfer TE, Overbach A, Greensher J. Unsafe playgrounds. Pediatrics 1979 12;64(6):962–963. 514729

[20] Abelsohn AR, Sanborn M. Lead and children: Clinical management for family physicians. Can Fam Physician 2010 06;56(6):531–535. 20547517 PMC2902938

[21] Centers for Disease Control. Lead. 2017; Available at: https://www.cdc.gov/nceh/lead/. Accessed December 12, 2017.

[22] U.S. Environmental Protection Agency. Tire Crumb Questions and Answers. 2017; Available at: https://www.epa.gov/chemical-research/tire-crumb-questions-and-answers. Accessed December 13, 2017.

[23] U.S. Consumer Product Safety Commission. Public playground safety handbook.: Government Printing Office; 2010.

[24] Mandzhieva SS, Minkina T, Pinskiy D, Bauer T, Sushkova S. The role of soil’s particle-size fractions in the adsorption of heavy metals. Eurasian Journal of Soil Science. 2014; 3: 197–205.

[25] Orroño DI and Lavado RS. Distribution of extractable heavy meatls in different soil fractions. Chemical Speciation and Bioavailability. 2009;21(3): 193–198.

[26] Knorr R. Preventing Childhood Lead Poisoning in Massachusetts. Massachusetts Department of Public Health. Available at: https://www.cityofboston.gov/images_documents/Robert%20Knorr,%20Preventing%20Childhood%20Lead%20Posioning%20in%20MA_tcm3-48543.pdf Accessed March 30, 2019.

[27] Mielke HW. Lead in New Orleans soils: New images of an urban environment. Environ Geochem Health 1994 12/01;16(3):123–128. 24197206 10.1007/BF01747908

[28] Schwarz K, Pickett STA, Lathrop RG, Weathers KC, Pouyat RV, Cadenasso ML. The effects of the urban built environment on the spatial distribution of lead in residential soils. Environmental Pollution 2012 4 2012;163(Supplement C):32–39. 22325428 10.1016/j.envpol.2011.12.003

[29] Mielke HW, Reagan PL. Soil is an important pathway of human lead exposure. Environ Health Perspect 1998 02;106:217–229. 10.1289/ehp.98106s1217 PMC1533263 9539015

[30] United States Environmental Protection Agency. Sources of Lead in Soil: A Literature Review. 1998; EPA 747-R-98-001a. Available at: https://www.epa.gov/sites/production/files/documents/r98-001a.pdf. Accessed March 30, 2019.

[31] United States Environmental Protection Agency. Lead Trends: National Trends in Lead Levels. Available at: https://www.epa.gov/air-trends/lead-trends. Accessed March 30, 2019.

[32] Coull B. Power Calculations and Study Design.

[33] City of Boston. Parks and Recreation Directory. 2014. Available at: https://www.cityofboston.gov/images_documents/Park%20Directory%20-%20June%202014_tcm3-44633.pdf

[34] Boston Public Health Commission. Health of Boston Report 2016–2017—Chapter 2: Social Determinants of Health, 2017; Available at: http://www.bphc.org/healthdata/health-of-boston-report/Documents/5_C2_SDH_16-17_HOB_final-5.pdf. Accessed March 30, 2019.

[35] MassDOT. MassGIS Data—Massachusetts Department of Transportation (MassDOT) Roads. June 2014. Commonwealth of Massachusetts. Available at: www.mass.gov/anf/research-and-tech/it-serv-and-support/application-serv/office-of-geographic-information-massgis/datalayers/eotroads.html

[36] Mass Administration and Finance. 2010 U.S. Census—Environmental Justice Populations. December 2012. Commonwealth of Massachusetts. Accessed at: www.mass.gov/anf/research-and-tech/it-serv-and-support/application-serv/office-of-geographic-information-massgis/datalayers/cen2010ej.html

[37] U.S. Geological Survey. Massachusetts State Data: Geogenic Soil Lead Concentrations. Avaiable at: https://www.epa.gov/superfund/usgs-background-soil-lead-survey-state-data#MA. Accessed December 12, 2017.

[38] Zhang JJ, Han IK, Zhang L, Crain W. Hazardous chemicals in synthetic turf materials and their bioaccessibility in digestive fluids. J Expo Sci Environ Epidemiol 2008 11;18(6):600–607. 10.1038/jes.2008.55 18728695

[39] Ginsberg G, Toal B. Human health risk assessment of artificial turf fields based upon results from five fields in Connecticut. Environmental and Occupational Health Assessment 2010:3–4.

[40] U.S. Environmental Protection Agency. Hazard Standards for Lead in Paint, Dust and Soil (TSCA Section 403). 2001.

[41] Nielsen JB, Kristiansen J. Remediation of soil from lead-contaminated kindergartens reduces the amount of lead adhering to children's hands. Journal Of Exposure Analysis And Environmental Epidemiology 2004 8/18;15:282. 10.1038/sj.jea.7500403 15316573

[42] Duggan MJ, Inskip MJ, Rundle SA, Moorcroft JS. Lead in playground dust and on the hands of schoolchildren. Sci Total Environ 1985 7;44(1):65–79. 4023696 10.1016/0048-9697(85)90051-8

[43] National Research Council. Bioavailability of Contaminants in Soils and Sediments: Processes, Tools, and Applications. Chapter 4. 2003. Available at: https://www.nap.edu/read/10523/chapter/6. Accessed March 30, 2019.

[44] Dixon SL, McLaine P, Kawecki C, Maxfield R, Duran S, Hynes P, et al The effectiveness of low-cost soil treatments to reduce soil and dust lead hazards: The Boston lead safe yards low cost lead in soil treatment, demonstration and evaluation. Environmental Research 2006 9 2006;102(1):113–124. 10.1016/j.envres.2006.01.006 16500641

[45] Carlisle J, Dowling K. Child-specific benchmark change in blood lead concentration for school site risk assessment. California Environmental Protection Agency: Sacramento, CA. 4 2007 Apr.

